# Fast Lithium Ion Conduction in Lithium Phosphidoaluminates

**DOI:** 10.1002/anie.201914613

**Published:** 2020-01-07

**Authors:** Tassilo M. F. Restle, Christian Sedlmeier, Holger Kirchhain, Wilhelm Klein, Gabriele Raudaschl‐Sieber, Volker L. Deringer, Leo van Wüllen, Hubert A. Gasteiger, Thomas F. Fässler

**Affiliations:** ^1^ Department of Chemistry Chair for Inorganic Chemistry with Focus on New Materials Technische Universität München Lichtenbergstraße 4 85747 Garching Germany; ^2^ Department of Chemistry and Catalysis Research Center Chair of Technical Electrochemistry Technische Universität München Lichtenbergstraße 4 85747 Garching Germany; ^3^ Department of Chemistry Chair of Inorganic and Metal-Organic Chemistry Technische Universität München Lichtenbergstraße 4 85747 Garching Germany; ^4^ Department of Physics University of Augsburg Universitätsstraße 1 86159 Augsburg Germany; ^5^ Department of Engineering University of Cambridge Cambridge CB2 1PZ UK; ^6^ Present address: Department of Chemistry University of Oxford Oxford OX1 3QR UK

**Keywords:** all-solid-state batteries, impedance spectroscopy, lithium, solid electrolytes, solid-state structures

## Abstract

Solid electrolyte materials are crucial for the development of high‐energy‐density all‐solid‐state batteries (ASSB) using a nonflammable electrolyte. In order to retain a low lithium‐ion transfer resistance, fast lithium ion conducting solid electrolytes are required. We report on the novel superionic conductor Li_9_AlP_4_ which is easily synthesised from the elements via ball‐milling and subsequent annealing at moderate temperatures and which is characterized by single‐crystal and powder X‐ray diffraction. This representative of the novel compound class of lithium phosphidoaluminates has, as an undoped material, a remarkable fast ionic conductivity of 3 mS cm^−1^ and a low activation energy of 29 kJ mol^−1^ as determined by impedance spectroscopy. Temperature‐dependent ^7^Li NMR spectroscopy supports the fast lithium motion. In addition, Li_9_AlP_4_ combines a very high lithium content with a very low theoretical density of 1.703 g cm^−3^. The distribution of the Li atoms over the diverse crystallographic positions between the [AlP_4_]^9−^ tetrahedra is analyzed by means of DFT calculations.

## Graphical abstract

**Lightweight and with fast lithium ion conduction**: Solid electrolytes with a fast ion conduction are crucial for all‐solid‐state batteries. The new material Li_9_AlP_4_ combines a high lithium content and a high ionic mobility of 3 mS cm^−1^ at 25 °C with a very low theoretical density of 1.703 g cm^−3^.

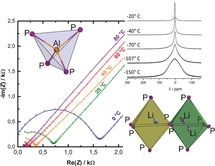

## Introduction

The development of advanced energy‐storage technologies plays a key role in realizing electric vehicles.^1^, ^2^, ^3^, ^4^ Next‐generation high‐energy‐density storage systems require low flammability, good electrochemical stability, and fast charging times. Li‐ion batteries based on organic electrolytes hinder the commercialization of long‐range electric vehicles. All‐solid‐state batteries (ASSBs) are promising candidates to overcome safety concerns of currently used Li‐ion batteries with flammable organic liquid electrolytes.^5^, ^6^, ^7^, ^8^ Replacing the organic liquid electrolyte by an inorganic solid‐state electrolyte (SSE), ASSBs offer high energy and power density, mechanical stability, and safety benefits.^9^, ^10^, ^11^, ^12^, ^13^, ^14^ However, ASSBs are limited by the slow ionic mobility of the SSE.^15^ Hence, the discovery, characterization, and optimization of lithium superionic conducting solid phases are among the main aspects of today's battery material research.^8^, ^16^, ^17^, ^18^ Despite the clear advantage of ASSBs, achieving Li‐ion conductivity in SSEs comparable to that in liquid electrolytes (>10 mS cm^−1^) is a demanding task.^9^ In the last decades, different crystalline materials have been proven to act as lithium conductors such as perovskite‐type structures,^19^, ^20^, ^21^, ^22^ lithium superionic conductor (LISICON)‐type structures,^23^, ^24^, ^25^, ^26^ thio‐LISICON‐type structures and thiophosphates,^27^, ^28^, ^29^, ^30^, ^31^, ^32^ sodium superionic conductor (NASICON)‐type structures,^33^, ^34^ garnet‐type structures,^35^, ^36^, ^37^ lithium argyrodites,^38^ lithium borohydrides,^39^ lithium nitrides,^40^, ^41^, ^42^ lithium hydrides,^43^ and lithium halides.^44^ The best lithium ion conductors currently known are rather complex systems such as Li_9.54_Si_1.74_P_1.44_S_11.7_Cl_0.3_ and Li_6+*x*_M_*x*_Sb_1−*x*_S_5_I (M=Si, Ge, Sn) with an ionic conductivity of 25 and 24 mS cm^−1^, respectively, outperforming the conductivity of liquid‐based electrolytes.^45^, ^46^ By increasing the carrier densities, changing the diffusion pathways of the mobile species, creating vacancies or increasing structural defects, the ionic conductivity can be further optimized.^8^, ^16^ An effective way to increase the carrier density is especially the aliovalent substitution of cations: for example, in Li_3_PS_4_ making a formal substitution of “P^5+”^ with “Ge^4+^” results in Li_3.25_Ge_0.25_P_0.75_S_4_ having a four times higher ionic conductivity.^28^

On the way to new candidates with good lithium ion conducting abilities, we recently have investigated lithium phosphidosilicates and phosphidogermanates.^47^, ^48^ The idea of replacing S^2−^ by P^3−^ enables the accommodation of even more lithium ions in the structures. Since our first report in 2016 on the aristo‐type Li_8_SiP_4_,^47^ also Li_14_SiP_6_ was established as good lithium ion conductor.^49^ Recently, we have also shown that the system can be extended to the heavier homologue germanium and that for Li_8_GeP_4_ two Li‐ion conducting modifications exist which display ionic conductivities of up to 8.6×10^−5^ S cm^−1^ at 298 K.^48^ Structurally, phosphidosilicates and phosphidogermanates are built up by [*Tt*P_4_]^8−^ tetrahedra, where *Tt* denotes the respective tetrel atom (Si, Ge). Relating to the building principles of oxidosilicates, thiosilicates, and thiophosphates, these tetrahedra can be formally condensed and covalently connected by sharing edges or corners; hence a large variety of structure motifs can be gained. The idea of phosphide‐based structures as ionic conductors results from the aliovalent substitution of [*Tt*S_4_]^4−^ tetrahedra, which are an key building block in sulfide‐based electrolytes, leading to the analogous complex anions based on phosphorus. Due to the resulting higher charge, [*Tt*P_4_]^8−^ tetrahedra allow for more lithium ions per formula unit for charge compensation when compared to [*Tt*S_4_]^4−^. In addition, the more negatively charged P^3−^ is more polarizable than S^2−^.

The coincidence of a higher charge carrier density due to more Li ions for charge compensation together with a large number of vacancies is considered as an important prerequisite for a higher lithium ion conductivity. Certainly, this aspect must be in line with a low activation energy for lithium mobility as it occurs in structures with an effective polyhedral connectivity.^50^ An aliovalent substitution of formal “Si^4+”^ by “Al^3+^” and formation of [AlP_4_]^9−^ instead of [SiP_4_]^8−^ tetrahedra allow for the presence of an even higher number of lithium ions and a strong influence on the Li occupation in voids. Ternary phases comprising the elements lithium, aluminum, and phosphorus were scarcely investigated. In the 1950s Juza et al. reported briefly the first lithium phosphidoaluminate, Li_3_AlP_2_, and described its structure as a strongly distorted fluorite‐type lattice in which P atoms form a close‐packed atom arrangement and Al and Li atoms are randomly distributed over the tetrahedral sites.^51^ Occupation of Li ions in octahedral sites was, however, not considered. Two further publications on Li_3_AlP_2_ were based on the poor structural characterization.^52^, ^53^

Here we report on the lithium‐richest representative of the phosphidoaluminates, obtained by formal aliovalent substitution of “*Tt* ^4+”^ by “Al^3+^” in Li_8_ *Tt*P_4_. The insertion of Al^3+^ leads to formally ninefold negatively charged [AlP_4_]^9−^ tetrahedra, resulting in an even higher lithium density per formula unit and a change in the spatial extent of the diffusion pathways. Due to the nature of the structure in which P atoms form a cubic‐close‐packing arrangement, there are still a high number of unoccupied octahedral sites present. We expect that an increase of the carrier density will also lead to an increase of the ionic conductivity. Indeed, only recently, ab initio simulations suggested that doping of the moderate lithium‐ion conductor Li_2_SiP_2_ with Al could enhance the ionic conductivity.^47^, ^54^

## Results and Discussion

### Synthesis and Crystal Structure of Li_9_AlP_4_

**Figure 1:**
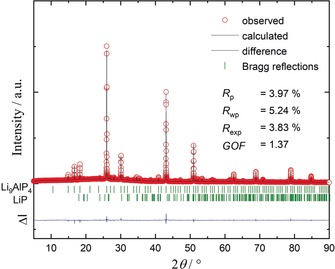
Rietveld analysis of the powder X‐ray diffractogram of Li_9_AlP_4_. The red dots indicates the observed intensities, the black line the calculated intensities, and the blue line the difference between both. Bragg positions are marked by green dashes.

Li_9_AlP_4_ was synthesized from the elements by a two‐step procedure. After ball‐milling of stoichiometric amounts of Li, Al and P, the powder mixture was annealed at 973 K for one day yielding almost phase‐pure Li_9_AlP_4_ with 2.3(1) wt % LiP impurities (see Figure 1). Complete data of the Rietveld refinement are given in the Supporting Information (Tables S1 and S2). Single crystals of Li_9_AlP_4_ were obtained by the direct reaction of the elements at 1073 K with a slight excess of P using a ratio of 9:1:4.2 (Li:Al:P). The resulting product contains besides Li_9_AlP_4_ also Li_3_P and TaP (see Figure S4). Energy‐dispersive X‐ray spectroscopy (EDX) investigations of single crystals show an Al/P ratio which is in very good agreement with the expected values (see Table S6). Li_9_AlP_4_ can also be synthesized starting from Li_3_P. However, again unreacted Li_3_P remains as a contamination (see Figure S5). According to EDX measurements, the single crystals were free of Ta. The details of the structure refinement of the single‐crystal X‐ray diffraction of Li_9_AlP_4_ are listed in Table 1 (further data are given in Tables S3 and S4 in the Supporting Information).

**Table 1:** Crystallographic data and refinement parameters of the SC‐XRD analysis of Li_9_AlP_4_.

	Empirical formula	Li_8.7(2)_AlP_4_	
	Formula weight [g mol^−1^]	210.93	
	Crystal size [mm^3^]	0.2×0.2×0.1	
	Crystal color	dark brown‐golden	
	Crystal shape	plate	
	*T* [K]	150(1)	
	Space group	$P\bar{4}3n$ (no. 218)	
	Unit cell dimension [Å]	*a*=11.852(1)	
	*V* [Å^3^]	1664.7(6)	
	*Z*	8	
	*ρ* (calc.) [g cm^−3^]	1.703	
	*μ* [mm^−1^]	0.908	
	*θ* Range [°]	2.430–32.494	
	Index range *hkl*	−17≤*h*≤17	
		−17≤*k*≤16	
		−17≤*k*≤12	
	Reflections collected	13 214	
	Independent reflections	1013	
	*R*_int_	0.0498	
	Reflections with *I*>2*σ*(*I*)	758	
	Data/restraints/parameter	1013/0/63	
	Absorption correction	empirical	
	Goodness‐of‐fit on *F* ^2^	1.277	
	Final *R* indices [*I*>2*σ*(*I*)]^[a,b]^	*R*_1_=0.026	
		*wR*_2_=0.067	
	*R* indices (all data)^[a,b]^	*R*_1_=0.041	
		*wR*_2_=0.080	
	Largest diff. peak and hole [e Å^−3^]	0.362/−0.417	
	Depository no.	CSD‐1962473

[a] $R_{1}=\sum \left|\left|F_{o}\right|-\left|F_{c}\right|\right|/\sum \left|F_{o}\right|$ . [b] $wR_{2}={\left[\sum w(F_{o}^{2}-{F_{c}^{2})}^{2}/\sum {w\left(F_{o}^{2}\right)}^{2}\right]}^{1/2}$ .

**Figure 2:**
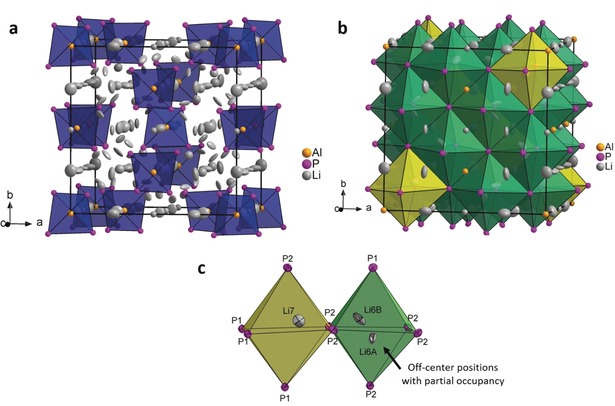
The crystal structure of Li_9_AlP_4_ as determined from experimental data. a) Unit cell of Li_9_AlP_4_. The [AlP_4_]^9−^ tetrahedra are highlighted in blue. Li^+^ ions are located in tetrahedral and octahedral voids; some of them exhibit partial occupations (Table S3). b) Unit cell with emphasis on the two crystallographically independent octahedral voids: polyhedra of Li6a/Li6b and Li7 are shown in green and yellow, respectively. c) Closeup of neighboring octahedral voids with partially occupied Li6a, Li6b, and Li7 positions. Li, Al, and P are depicted in gray, orange, and purple, respectively (displacement ellipsoids set at 90 % probability at 150 K).

According to single‐crystal and powder X‐ray structure determination and refinement, Li_9_AlP_4_ crystallizes in the cubic space group $P\bar{4}3n$ (no. 218). The structure of Li_9_AlP_4_ is built up of isolated [AlP_4_]^9−^ tetrahedra surrounded by Li^+^ ions (Figure 2 a). The AlP_4_ tetrahedra are slightly distorted with P‐Al‐P angles ranging from 110.89(2)° to 108.77(2)° in comparison to the ideal tetrahedron angle of 109.74°. The Al–P bonds at 2.425(1) and 2.433(1) Å are longer than the Al–P bonds observed in compounds with connected AlP_4_ tetrahedra like AlP (2.360(1) Å), Na_3_AlP_2_ (2.376(4) Å), and Sr_3_Al_2_P_4_ (2.377(3) Å–2.417(2) Å).^55^, ^56^, ^57^

For the discussion of the coordination polyhedra of Li atoms, the structure is considered as a distorted cubic‐face‐centered packing of P atoms with Li and Al atoms filling voids in between and Wyckoff positions giving the multiplicity of the site. With *Z*=8, there are 32 P atoms per unit cell resulting in 64 tetrahedral and 32 octahedral voids. The atoms Al1 and Al2 occupy 1/8 of the tetrahedral voids on Wyckoff positions 2a and 6d, respectively. We like to point out that the Al atoms are fully ordered, and no mixed occupancy of Al and Li occurs, in contrast to the mixed Li/Si occupancy in the Li‐rich phase Li_14_SiP_6_.^49^ The remaining tetrahedral voids in the title compound are occupied by Li atoms as follows: Li1, Li4, and Li5 (on Wyckoff positions 6b, 12f, and 24i, respectively) are fully occupied, whereas Li2 and Li3 (on Wyckoff positions 6c and 8e, respectively) are partially occupied with side occupation factors 0.50(5) and 0.71(4), respectively, based on single‐crystal structure determination data. Hence, the overall occupation of tetrahedral positions by lithium is 91 %.

The occupation of the 32 larger octahedral voids reveals two interesting aspects. The voids are partially occupied and in addition, we see for the first time that the Li atoms are not necessarily located at the center of the P_6_ octahedra (Figure 2 c). The 32 octahedral voids can be distinguished by two crystallographically different Wyckoff positions that are both partially occupied with Li: Li7 on Wyckoff position 8e with site occupation factor (*sof*) 0.25(4) and a split position of Li6a and Li6b on Wyckoff position 24i with *sof* 0.46(2) and *sof* 0.23(2), respectively (Figure 2 b). The overall occupation of the octahedral voids, therefore, is 58 %.

Due to the good quality of the crystallographic data it becomes obvious that Li6a, Li6b, and Li7 are not located in the centers of the octahedra, and the data also allow the determination of split positions in one octahedral void as anticipated for other lithium ion conducting materials that have Li ions in octahedral environments, as in garnets (Li_7_La_3_Zr_2_O_12_) and sulfide‐based materials (Li_10_GeP_2_S_12_).^58^, ^59^ The refined positions of the octahedrally coordinated Li ions are shifted towards the triangular faces of the octahedra, where the face‐sharing LiP_4_ tetrahedra of the partially occupied Li sites Li2 and Li3 are located.

All Li atoms exhibit large displacement ellipsoids (Figure 2 a) which points towards a static or thermal displacement indicative of a high lithium ion mobility. All atomic positions except Li1 were refined anisotropically. Interestingly, the octahedral positions Li6a and Li6b exhibit ellipsoids that point towards the center of the triangular faces of the neighboring tetrahedral voids that are only partially filled with Li2 and Li3 (Figure 2 b).

The high structural diversity of the Li atoms including disorder and split positions reflects the possibility of high lithium ion mobility. Li–P distances in the tetrahedral voids range from 2.50(1) to 2.73(1) Å, and in the octahedral voids they range from 2.56(1) to 3.46(1) Å. The bond lengths are similar to those in other ternary phases containing Li and P, such as Li_8_SiP_4_, Li_2_SiP_2_, Li_10_SiP_2_, and Li_3_Si_3_P_7_.^47^, ^60^

According to the valence rules, Li_9_AlP_4_ is an electron‐precise compound and can be described as (Li^+^)_9_[AlP_4_]^9−^, with formally two negative charges for the P atoms and one negative charge for the Al atom, balanced by nine Li^+^ ions.

### DFT Calculations

**Figure 3:**
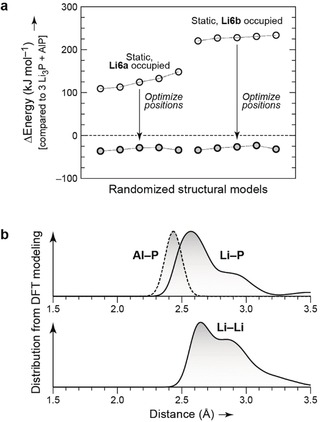
a) DFT‐computed energy of 10 randomized structural models, compared to that of the competing binary phases (3 Li_3_P + AlP) which are set as energy zero. The models are ordered by ascending (unrelaxed) energy; lines connecting symbols are only guides to the eye. Static computations (using the experimental structure; open circles) indicate a clear preference for the Li6a over the Li6b site. DFT optimization of these structures (arrows) yields an ensemble of models that are all more favorable than the competing binaries and are essentially degenerate in energy (see the Supporting Information for further details). b) Distribution of interatomic distances in randomized structural models of Li_9_AlP_4_, optimized using DFT, as detailed in the Supporting Information. Kernel density estimates (“smoothed histograms”) with a bandwidth of 0.05 Å are shown to characterize all relevant contacts collected over 10 structural models, which approximate the real structure within the limits of theory.

DFT computations based on the experimentally derived structural information were performed to serve a double purpose: first, to corroborate the refined structural model obtained from experiments; second, to obtain information about stability trends and the coordination of individual atoms in the presence of crystallographic disorder. We constructed a series of discrete atomistic models which approximate the disordered structure (making simplifications as detailed in the Supporting Information). The energetic stability of the new compound as compared to competing phases is analyzed according to the line in the ternary composition (Gibbs) diagram. Since Li_9_AlP_4_ is located on the line between the binaries Li_3_P and AlP (Figure S6), it is straightforward to inquire the formation energy according to 3 Li_3_P + AlP (=Li_9_AlP_4_) and to assess whether the title compound is stable with regard to the constituent binaries. The computed energies of ten randomized structural models were compared to those of the competing binary phases (3 Li_3_P + AlP) which are set as energy zero. The formation of the ternary title compound from the binaries is energetically favored by approximately 30 kJ mol^−1^ (Figure 3 a and the Supporting Information), even without taking configurational entropy into account (which will further stabilize the ternary phase, as there are no partially occupied crystallographic positions in either Li_3_P or AlP).

In addition to the energetic stability, having an ensemble of computationally optimized structural models allows us to quantify the distribution of interatomic distances and to compare with experimental results. Smoothed histograms over all ten structures are given in Figure 3 b. The computed Al–P distances in the relaxed structures are 2.44(4) Å, with a narrow distribution indicating the rigidity of the tetrahedral AlP_4_ units. The Li–P distributions peak at 2.6 Å, but include a substantial amount of larger distances, reflecting the more disordered nature of the Li atoms which are often shifted away from the centers of the octahedral voids. The distribution of Li⋅⋅⋅Li distances in the relaxed structures spans a broad range, starting at ≈2.4 Å, peaking at ≈2.6 Å, and becoming significantly less pronounced beyond 3.0 Å, in good agreement with the experimental observations (Table S5).

### Impedance Spectroscopy

**Figure 4:**
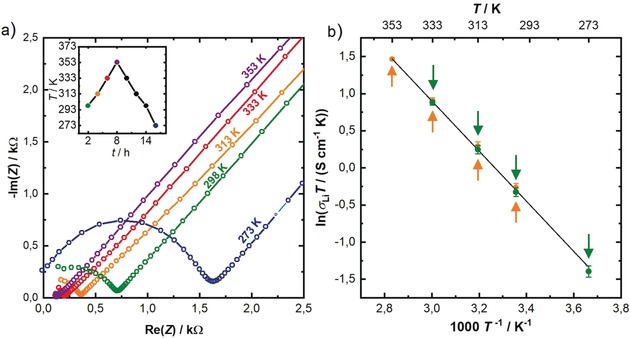
a) Nyquist plot of Li_9_AlP_4_ measured under blocking conditions, with spectra recorded at temperatures between 273 and 353 K during a heating cycle, acquired in a sequence described by the color‐coded points marked in the temperature profile that is shown in the inset (note that the 273 K data were acquired at the end of the first heating and cooling cycle). b) Arrhenius plot of the product of conductivity and temperature (*σ*_Li_ *T*) obtained in the heating (orange) as well as in the cooling (green) branch, with error bars for each based on the standard deviation from independent measurements with three cells; the shown linear fit through both branches was used to obtain the activation energy $E_{A}^{\mathrm{PEIS}}$ . Since the differences of the average (*σ*_Li_ *T*) values obtained during heating vs. cooling are very small; they are marked by the orange and green arrows, respectively.

The lithium ion conductivity of Li_9_AlP_4_ was determined from impedance measurements in a blocking electrode configuration. Impedance spectra at different temperatures (273, 298, 313, 333, and 353 K according to the temperature profile shown in the inset) are displayed in Figure 4 a and feature a semicircle at high frequencies and a low‐frequency tail. The semicircle can be described as parallel circuit element of a resistor and a constant phase element (*R*/*Q*), with *R* representing both intragrain and grain boundary contributions to the lithium ion transport which could not be resolved, and thus only the total ionic resistance of the sample could be determined. For the constant phase element, the fit of the data acquired at 298 K resulted in *α* values of ≈0.87 and *Q* parameters with a value of ≈17×10^−9^ F s^(*α*−1)^; the ionic conductivity was determined to be σ_Li_(Li_9_AlP_4_)=(3.03±0.16)×10^−3^ S cm^−1^ at 298 K (obtained from three independently measured cells). The activation energy for lithium ion transport (Figure 4 b) was investigated by temperature‐dependent impedance measurements in the range from 273–353 K, yielding *E*_A_^PEIS^ of 28.5±0.8 kJ mol^−1^ (≈0.29 eV); this was determined from three independently measured cells, using the *σ*_Li_ *T* values of only the first heating and cooling cycle of each sample. This activation energy is in very good agreement with the value obtained by NMR spectroscopy (shown below). The temperature ramp of a heating and cooling cycle is displayed in the inset of Figure 4 a. Colored dots indicate at which temperatures PEIS measurements were performed. In this context it should be mentioned that conductivities (and thus the product *σ*_Li_ *T*) for heating and cooling differ by less than 6 %. Error bars are calculated separately for heating and cooling steps by taking the mean of three independent measurements obtained from three cells. DC polarization measurements in the range from 50–150 mV reveal an electronic conductivity of (2.0±0.8)×10^−7^ S cm^−1^ at 298 K (based on the standard deviation of two cells).

### ^6^Li and ^7^Li NMR Spectroscopy

**Figure 5:**
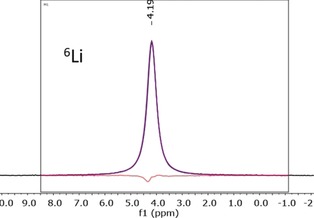
^6^Li MAS NMR measurement of Li_9_AlP_4_. The inset shows the region of data fitting. The black, pink, and red lines indicate the experimental and calculated signal and the difference line, respectively. Full width at half maximum is 19.2 Hz.

The ^6^Li MAS NMR spectrum at room temperature shows one signal at a chemical shift of 4.19 ppm (Figure 5). The fitting of a generalized Lorentzian function to the experimental data proves the presence of only one signal as observed before for related compounds.^47^, ^48^ Since the shifts of Li atoms in tetrahedral and octahedral voids are not distinguishable, it can be assumed that all lithium ions are mobile at room temperature.

Static ^7^Li NMR spectra were recorded as a function of temperature to study the dynamic behavior of the lithium ions. The central transition of the *I*=3/2 nucleus ^7^Li experiences a broadening only from the homo‐ (^7^Li–^7^Li) and heteronuclear (here: ^7^Li–^31^P) dipolar couplings. Since both types of interactions scale with the second Legendrian (3 cos^2^ *β*−1), any dynamic process should produce a (partial) averaging of the orientational dependence and hence entail a narrowing of the NMR signal.

**Figure 6:**
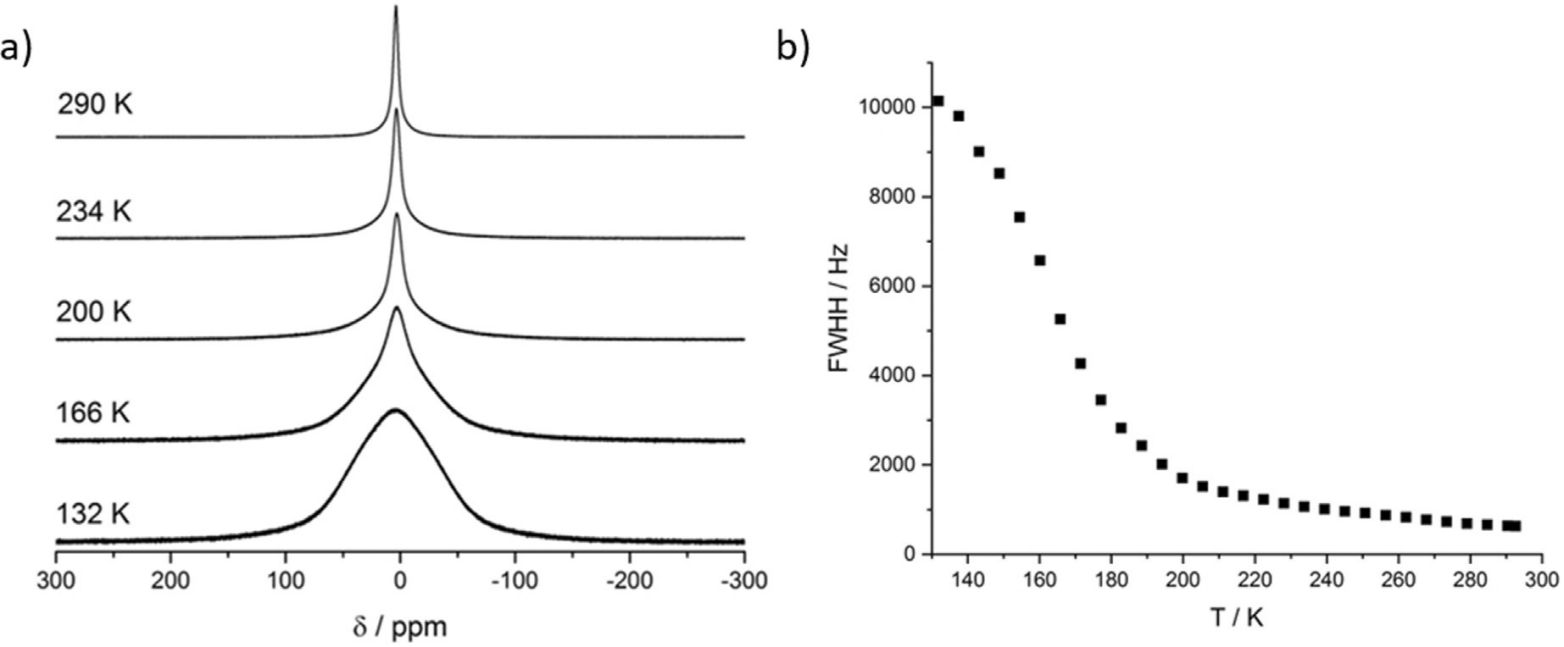
a) Temperature‐dependent evolution of the ^7^Li lineshape recorded in the temperature range from 132 to 290 K. b) Temperature‐dependent linewidth of the ^7^Li signal (full width at half height) recorded in the temperature range from 132 K to room temperature.

At 290 K, the static ^7^Li spectrum (Figure 6 a top) displays a homogeneous Lorentzian shape with a linewidth of 640 Hz. Upon cooling, the signal gradually broadens, developing a Gaussian line shape with a linewidth of 10.1 kHz (Figure 6 a bottom). At intermediate temperatures (166 K and 200 K), the line shape indicates some heterogeneity, which may be related to a distribution of activation energies for the Li ions occupying eight distinct crystallographic positions. Individual contributions from the different Li sites in the crystal lattice could not be resolved. For temperatures >234 K a homogeneous line shape is observed, indicating all Li ions are mobile.

The temperature‐dependent evolution of the linewidth (FWHH; full width at half height) of the ^7^Li NMR signal is shown in Figure 6 b. A rough estimation of the activation energy of lithium motion is possible employing the empirical Waugh–Fedin relation *E*_A_ (kJ mol^−1^)=0.156 *T*_onset_ (K).^61^ As the onset temperature, we identified the temperature at which the linewidth is given by (*ν*_rigid lattice_−*ν*_mot. narrowing_)/2. This leads to an approximate onset temperature of 160 K which translates to an activation energy of 25 kJ mol^−1^.

## Conclusion

Recently, we reported the synthesis and characterization of Li_14_SiP_6_, for which we had optimized the ionic conductivity (≈1 mS cm^−1^) in lithium phosphidotetrelates by increasing the amount of lithium compared to Li_8_SiP_4_.^49^ With Li_9_AlP_4_ we now show that these materials can be further optimized, namely by full aliovalent replacement of the tetrelate atom. Compared to the ionic conductivities in Li_8_SiP_4_ (4.5×10^−5^ S cm^−1^) and α/β‐Li_8_GeP_4_ (1.8×10^−5^ S cm^−1^, 8.6×10^−5^ S cm^−1^), via aliovalent substitution by aluminum and the associated higher lithium content in the structure alongside with a change in the distribution of vacancies (Table 2), the conductivity in Li_9_AlP_4_ is strongly increased up to 3.0(2) mS cm^−1^ at room temperature. Concomitantly, the activation energy determined by impedance measurements drops significantly from 42 (α‐Li_8_GeP_4_), 39 (Li_8_SiP_4_), and 38 kJ mol^−1^ (β‐Li_8_GeP_4_) to 29 kJ mol^−1^ in Li_9_AlP_4_.

**Table 2:** Overview of lithium occupancy, cell volume, lithium ion mobility at room temperature, and Li–P distances in Li_9_AlP_4,_ β‐Li_8_GeP_4_, α‐Li_8_GeP_4_, Li_8_SiP_4_, and Li_14_SiP_6_. Data for the latter four are taken from the literature.^47^, ^48^, ^49^

	Li_9_AlP_4_		β‐Li_8_GeP_4_	α‐Li_8_GeP_4_		Li_8_SiP_4_	Li_14_SiP_6_≡Li_9.33_Si_0.66_P_4_	
	Wyck.	*sof*	*sof*	Wyck.	*sof*	*sof*	Wyck.	*sof*
Tetra‐ hedral voids	6b	1	0.78(3)	8c	1	1	8c	0.92(1)
	6c	0.50(5)	1	24d	1	1		
	8e	0.71(4)	1	24d	1	1		
	12f	1	1					
	24i	1	0.887(9)					
Octa‐ hedral voids	24i	0.46(2)	0.502(9)	4a	1	1	4b	0.50(1)
	24i	0.23(2)	–	24d	–	0.1666		
	8e	0.25(4)	–	4b	1	–		
*V* [Å^3^] at RT		1674.22(2)	1631.75(1)		1643.88(2)	1603.06(2)		209.507(1) ≡1676.06(1)
σ_Li_ at RT		3 mS cm^−1^	8.6×10^−5^ S cm^−1^		1.8×10^−5^ S cm^−1^	4.5×10^−5^ S cm^−1^		1 mS cm^−1^
Li–P *d*_tet_ [Å]		2.50(1)–2.73(1)	2.50(1)–2.69(1)		2.47(2)–2.91(1)	2.51(1)–2.80(1)		2.57(1)
Li–P *d*_oct_ [Å]		2.56(1)–3.46(1)	2.82(2)–3.05(2)		2.884(1)–3.028(4)	2.86(1)–3.08(1)		2.97(1)

Li_9_AlP_4_ is easily accessible via ball‐milling and crystallizes in the cubic space group $P\bar{4}3n$ (no. 218). First‐principles computations suggest that Li_9_AlP_4_ is approximately 30 kJ mol^−1^ more stable than the constituent binary phosphides. The structure of Li_9_AlP_4_ shows a close relationship to the recently characterized lithium ion conductors Li_8_SiP_4_, α‐Li_8_GeP_4_, and β‐Li_8_GeP_4_. However, in Li_9_AlP_4_ the highly negatively charged, isolated [AlP_4_]^9−^ tetrahedra make it possible to accommodate a higher number of lithium ions per formula unit than in the phosphidotetrelates and therefore the new compound shows a much better Li ion conductivity. Whereas Li_8_GeP_4_ adopts both the α modification (space group $Pa\bar{3}$ , no. 205) at lower temperature and the β modification at higher temperatures (space group $P\bar{4}3n$ , no. 218), Li_9_AlP_4_ crystallizes exclusively in analogy to the β modification. These phosphidosilicates, ‐germanates, and ‐aluminates can be described as a close packing of P atoms with four octahedral and eight tetrahedral voids per formula unit. Consequently, Al, Si, and Ge atoms occupy 1/8 of the tetrahedral voids forming covalently connected Al–P, Si–P, and Ge–P bonds, respectively, and are located on fixed fully occupied positions in the structure. The series of compounds now give insight into the occupancy of the lithium atoms which varies according to Table 2. By comparing the lithium content, tetrahedral voids are slightly less occupied, whereas in the octahedral voids more lithium is found in Li_9_AlP_4_ compared to the situation in β‐Li_8_GeP_4_: in Li_9_AlP_4_ 90 % of the tetrahedral and 58 % of the octahedral voids are occupied compared to 93 % and 38 %, respectively, in β‐Li_8_GeP_4_. At a higher lithium content, the lithium ions are more evenly distributed over the tetrahedral and octahedral voids which might mainly result from a lower average electrostatic repulsion. The here observed decrease of lithium content in the tetrahedral and the increase of lithium content in the octahedral voids in the presence of more lithium is in accordance with the observation made by aliovalent substitution in garnets and was well investigated by Cussen.^62^ Moreover, this increased population of octahedral voids contributes likely to the energy landscape flattening, as also seen in further electrolytes like in Li_7_La_3_Zr_2_O_12_ and argyrodites.^58^, ^63^ We found that the displacement of the Li^+^ position from the center of the octahedral voids increases with an increasing lithium content per formula unit. And, as also observed in garnets, this stronger displacement correlates with a higher ion mobility. In Li_9_AlP_4_ we found, for the first time in a phosphide‐based material, a split position in the octahedral voids. Both positions are close to a triangular plane of the octahedra, supporting the hypothesis of lithium diffusion via face‐sharing tetrahedra and octahedra. Lithium migration network analysis in phosphide‐based materials like β‐Li_8_GeP_4_, has already suggested that lithium diffusion is favored via face‐sharing tetrahedra and octahedra.^48^ Interestingly, the split lithium positions in an octahedral environment, as observed here, has also been found in other fast ion conductors such as sulfide‐based electrolytes and garnets: In Li_10_GeP_2_S_12_ (LGPS), one Li atom (Wyckoff position 8g) exhibits a split position in a slightly distorted S_6_ octahedron,^59^ and in Li_7_La_3_Zr_2_O_12_ (LLZO), one Li atom (Wyckoff position 96h) located in a strongly distorted O_6_ octahedron appears with a split position as well.^58^

A change of the metal atom and a higher lithium concentration compared to the previously known compounds (Table 2) results in an extraordinarily high ionic conductivity of 3 mS cm^−1^ at room temperature, a very low activation energy of 29 kJ mol^−1^ (≈0.29 eV), and a roughly 4 orders of magnitude lower electronic conductivity of 2.0×10^−7^ S cm^−1^ at room temperature for the title compound. This high ionic conductivity is achieved by the accommodation of more lithium ions in the octahedral voids. One further benefit of Li_9_AlP_4_ is its very low density of ≈1.7 g cm^−3^, which makes the compound very attractive for applications in all‐solid‐state batteries.

Even though the Li content itself is not a decisive parameter for increasing the Li mobility, the possibility of aliovalent substitution of either Li_9−*x*_ *Tr*_1−*x*_ *Tt_x_*P_4_ (*Tr*=triel element) allows for manifold optimization possibilities. Therefore, we will further investigate the tetrahedral and octahedral occupancy in solid solutions Li_9−*x*_ *Tr*_1−*x*_ *Tt_x_*P_4_. Further studies will also focus on the thermal behavior and the electrochemical stability of this material. For Li_9_AlP_4_, temperature‐dependent powder neutron diffraction measurements in combination with MEM calculations are scheduled to localize the diffusion pathways.

## Conflict of interest

The authors declare no conflict of interest.

## Supporting information

As a service to our authors and readers, this journal provides supporting information supplied by the authors. Such materials are peer reviewed and may be re‐organized for online delivery, but are not copy‐edited or typeset. Technical support issues arising from supporting information (other than missing files) should be addressed to the authors.

Supplementary
