# Supplementary material for: Fast Lithium Ion Conduction in Lithium Phosphidoaluminates
Source: Angew Chem Int Ed Engl. 2020 Jan 7;59(14):5665–74. doi: 10.1002/anie.201914613 (PMC7154659; doi:10.1002/anie.201914613)
Supplement: Supplementary file 1 — Supplementary [file ANIE-59-5665-s001.pdf]

Angewandte
Eine Zeitschrift der Gesellschaft Deutscher Chemiker
Chemie

Supporting Information

Fast Lithium Ion Conduction in Lithium Phosphidoaluminates

*Tassilo M. F. Restle, Christian Sedlmeier, Holger Kirchhain, Wilhelm Klein, Gabriele Raudaschl-Sieber, Volker L. Deringer, Leo van Wüllen, Hubert A. Gasteiger, and Thomas F. Fässler**

anie_201914613_sm_miscellaneous_information.pdf

Supporting Information

Experimental Section

All steps of synthesis and sample preparations were carried out inside an argon-filled glove box (*MBraun*, *p*(H_2O), *p*(O_2) < 0.1 ppm). Prior to use, lithium (Li, rods, Rockwood Lithium, > 99 %) was cleaned from oxide layers. Aluminium (Al, granules and powder, ChemPur, 99.99 %) and phosphorus (P, powder, Sigma-Aldrich, 97 %) were used without any further purification. If not mentioned differently, aluminium granules were used for synthesis. Lithium phosphide (Li_3P) was synthesized according to H. Eickhoff *et al.*[1]

Synthesis of Li_9AlP_4. Li_9AlP_4 was synthesized from the elements via ball milling and subsequent annealing. Lithium (658.4 mg, 93.9 mmol, 9 equiv.), aluminium (281.5 mg, 10.4 mmol, 1 equiv.) and phosphorus (1332.8 mg, 41.7 mmol, 4 equiv.) were ball milled (Retsch PM100 Planetary Ball Mill) for 36 h at 350 rpm with rest periods (every 10 min for 3 min) using a WC milling set (50 mL jar and with 3 balls with a diameter of 1.5 cm). The obtained dark-red mixture was pressed into pellets with a diameter of 13 mm for 30 s at 5 t using a hydraulic press (Specac Atlas 15T). The fragmented pellets were filled into graphitized quartz ampoules (8 mm diameter) which were sealed under vacuum with an oxyhydrogen burner. The sealed ampoules were heated in a chamber furnace (Nabertherm P 330) with 5 $K{\cdot}min^{-1}$ up to 973 K, dwelled for 24 h, and subsequently cooled with 0.5 $K{\cdot}min^{-1}$ to room temperature. After pestling of the pellets, a brown-red powder was obtained.

Brown-golden coloured flaky single crystals were obtained by the reaction of the elements with a slight excess of phosphorus in a tantalum ampoule. Lithium (72.0 mg, 10.3 mmol, 8.9 equiv.), aluminium (31.0 mg, 1.1 mmol, 1.0 equiv.) and phosphorus (153.0 mg, 4.8 mmol, 4.2 equiv.) were filled into a tantalum ampoule. The ampoule was sealed in an electric arc furnace (Edmund Bühler MAM1), enclosed in a quartz reaction container under vacuum and subsequently heated with 5 $K{\cdot}min^{-1}$ up to 1073 K, dwelled for 24 h, and cooled with 0.5 $K{\cdot}min^{-1}$ to room temperature in a tube furnace (HTM Reetz Loba 1200-42-600-1-OW with a EUROTHERM S 14083 temperature controller), yielding a dark-ochre, crystalline product.

Li_9AlP_4 was also obtained by using Li_3P as lithium source. For this, lithium phosphide (82.4 mg, 1.6 mmol, 3 equiv.) and phosphorus (16.9 mg, 0.53 mmol, 1 equiv.) were homogenized in a mortar, mixed with aluminium powder (14.3 mg, 0.53 mmol, 1 equiv.) and pressed to a pellet with a diameter of 13 mm for 30 s at 5 t using a hydraulic press. The pellet was placed into a niobium ampoule. The ampoule was closed in an electric arc furnace and treated accordingly to the synthesis from the elements. After pestling of the pellet a brown-red powder was obtained.

Powder X-ray diffraction. For Powder X-ray diffraction (PXRD) measurements, the samples were ground in an agate mortar and sealed inside 0.3 mm glass capillaries. PXRD measurements were performed at room temperature on a STOE Stadi P diffractometer equipped with a Ge(111) monochromator for Cu $K_{\alpha 1}$ radiation (λ = 1.54056 Å) and a Dectris MYTHEN DCS 1K solid-state detector. The raw powder data were processed with the software package WinXPOW.[2]

Rietveld refinement. Rietveld refinements were performed with TOPAS V6.[3] The single crystal solution was used as the structural model. All cell parameters and the atom positions for Al and P and Li were refined freely. Isotropic displacement parameters were refined freely for P and coupled for Al, i.e. for both Al atom positions a joint displacement parameter was assigned. Isotropic displacement parameters for lithium atoms were fixed according to the values obtained by SCXRD. Site occupancy factors for lithium atoms were refined with the constraint according to the single crystal data which exhibits that an overall occupancy of lithium atoms with a distance lower than 2 Å does not exceed 100 %. The Rietveld analysis was performed for data taken at room temperature (further details are in Table S1 and S2). The difference in lattice parameters from Rietveld analysis (a = 11.87419(5) Å) compared to the single crystal (a = 11.852(1) Å) results from different measuring temperatures (PXRD at 293 K and SC-XRD at 150 K). The Rietveld analysis gives less reliable occupation factors for the lithium atoms (the occupancies of Li2/Li6a and Li3/Li7 had to be coupled during refinement). Therefore, single crystal data was chosen to be discussed in the results and discussion part.

Single crystal structure determination. A single crystal of Li_9AlP_4 was sealed in a 0.3 mm glass capillary. The single crystal X-ray diffraction (SCXRD) measurement was carried out on a STOE Stadivari diffractometer equipped with a Ge(111) monochromator, Mo $K_{\alpha 1}$ radiation (λ = 0.71073 Å) source and a DECTRIS PILATUS3R 300 K detector. The structure was solved by Direct Methods (SHELXS) and refined by full-matrix least-squares calculations against F^2 (SHELXL).[4]

Energy-Dispersive X-ray Spectroscopy (EDX). EDX spectra of Li_9AlP_4 single crystals were measured on a Hitachi TM-1000 Tabletop (15 kV) scanning electron microscope equipped with an energy dispersive X-ray analyzer (SWIFT-ED-TM). The samples were mounted onto an aluminium stub using graphite tape. To exclude aluminium impurities of the stub in the EDX spectra, measurements of the graphite tape on the aluminium stub without any samples were performed resulting in no aluminium signal. Three measurements were performed, and the average was calculated.

NMR spectroscopy. Magic angle spinning (MAS) NMR spectroscopy was carried out on a Bruker Avance 300 NMR device operating at 7.04 T by the use of a 4 mm ZrO_2 rotor. The resonance frequency is 44.17 MHz for ^6Li. The rotational frequency was 15 kHz. The MAS spectrum has been acquired at room temperature with a relaxation delay of 5 s and 120 scans. The spectra were referenced to LiCl (1 M,

aq) and LiCl (s) offering chemical shifts of 0.0 ppm and -1.15 ppm, respectively. The spectra were recorded using single-pulse excitation.

Low-temperature ^{7}Li measurements have been performed at a 7.04 T Bruker Avance 3 spectrometer equipped with a 4mm-MAS-WVT-Probe. The resonance frequency of ^{7}Li at this field strength is 116.64 MHz. For temperature-dependent static ^{7}Li spectra, the sample has been enclosed in a glass-ampoule to avoid contact with air and moisture. A saturation comb has been used prior to data acquisition, and a relaxation delay between 30 s and 1 s and 4 repetitions has been typically used. The spectra were referenced to a 9.7 molar LiCl-solution. Temperature calibration has been performed by measuring the temperature-dependent chemical shift of lead nitrate, $Pb(NO_3)_2$, which has been enclosed in a glass ampoule as well.

Impedance Spectroscopy and DC Conductivity Measurements

The ionic conductivity of Li_9AlP_4 was determined by electrochemical impedance spectroscopy (EIS) in an in-house designed cell. The setup consists of two stainless-steel current collectors, a stainless-steel casing, a PEEK tube, hardened stainless-steel dies and pistons comprising a gasket for tightening the cell as well as six screws for fixing the cell. Powdered samples of Li_9AlP_4 (500 mg) were placed between two 8 mm dies, and the screws were fastened with a torque of 30 Nm, compressing the samples to 89% of the crystal density. The setup is described in detail by Strangmüller *et al.*[5] For the determination of the compressed pellet thickness, six holes in a symmetric configuration were drilled into the current collectors, and the distance in between was measured using a precision caliper. Impedance spectra were recorded on a Bio-Logic potentiostat (SP-300) in a frequency range from 3 MHz to 50 mHz at a potentiostatic excitation of ±50 mV. Data were treated using the software EC-Lab (V 11.27). The measurements were performed in an Ar-filled glove box between 299 – 300 K. The electronic conductivity was determined with the same setup using a potentiostatic polarization procedure, applying voltages of 50, 100 and 150 mV for 15 h each. For determining the activation energy of lithium ion conduction, the cell temperature was set to 273, 298, 313, 333, and 353 K using a climate chamber (ESPEC, LU-114). The exact temperature profile is described in the Supporting Information as well as in Figure S7a. Prior to EIS measurements, the cell was allowed to rest for 120 min for thermal equilibration. EIS measurements were performed at both heating and cooling cycles. Temperature-dependent measurements were carried out outside the glove box, and the piston of the cell was additionally greased to ensure a tight sealing of the cell from the ambient environment.

First-principles computations

Model density-functional theory (DFT) computations were carried out to assess the distribution of atoms in the disordered structure and to estimate the energetic stability of the newly synthesized

phase. All DFT computations were carried out with CASTEP 8.0,[6] using the PBEsol exchange–correlation functional[7] and on-the-fly generated pseudopotentials. Details of how the discrete structural models are constructed and of the computational parameters are provided as Supporting Information.

Rietveld Data

Table S1. Crystallographic data of Li_9AlP_4 obtained from powder diffraction data by Rietveld refinement.

Empirical formula	$Li_{8.7(2)}AlP_4$
Formula weight / g·mol^{-1}	210.93
T/K	293
Powder color	red-brown
Crystal system	cubic
Space group	$P\bar{4}3n$ (Nr. 218)
Unit cell dimension	
a / Å	11.87419(5)
V / Å^3	1674.22(2)
Z	8
ρ (calc.) / g·cm^{-3}	1.67537
λ / Å	1.54060
Θ Range / °	4.957 - 90.037
R_p	0.0397
R_{wp}	0.0524
R_{exp}	0.0383
GOF	1.37
depository no.	CSD-1962474

Table S2. Atomic coordinates, isotropic displacement parameters and site occupancy factors (sof) for Li_9AlP_4 from Rietveld analysis.

**Atom**	**Wyck.**	***x***	***y***	***z***	U_{iso}	***sof***
Al1	2a	0	0	0	0.010(1)	
Al2	6d	½	¼	0	0.010(1)	
P1	8e	0.1204(5)	0.1204(5)	0.1204(5)	0.023(5)	
P2	24i	0.3758(2)	0.3683(2)	0.1162(4)	0.007(1)	
Li1	6b	½	½	0	0.008	
Li2	6c	¼	½	0	0.02	0.54(2)
Li3	8e	0.243	0.243	0.243	0.018	0.71(3)
Li4	12f	0.2562	0	0	0.028	
Li5	24i	0.498	0.2596	0.2530	0.026	
Li6a	24i	0.3363	0.583	0.0901	0.015	0.46(2)
Li6b	24i	0.334	0.158	0.162	0.014	0.21(2)
Li7	8e	0.340	0.340	0.340	0.02	0.29(3)

Single Crystal Data

Table S3. Atomic coordinates and site occupancy factors (sof) for Li_9AlP_4 from SC-XRD measurement.

Atom	Wyck.	*x*	*y*	*z*	*sof*	U_{iso}
Al1	2a	0	0	0		0.008(1)
Al2	6d	½	¼	0		0.0075(6)
P1	8e	0.11811(8)	0.11811(8)	0.11811(8)		0.0068(4)
P2	24i	0.37701(5)	0.36645(7)	0.11601(6)		0.0074(2)
Li1	6b	½	½	0		0.008(4)
Li2	6c	¼	½	0	0.50(5)	0.02(1)
Li3	8e	0.243(1)	0.243(1)	0.243(1)	0.71(4)	0.018(5)
Li4	12f	0.2562(9)	0	0		0.028(5)
Li5	24i	0.498(1)	0.2596(8)	0.2530(4)		0.026(4)
Li6a	24i	0.3363(9)	0.583(1)	0.0901(9)	0.46(2)	0.015(3)
Li6b	24i	0.334(2)	0.158(2)	0.162(2)	0.23(2)	0.014(6)
Li7	8e	0.340(2)	0.340(2)	0.340(2)	0.25(4)	0.02(1)

Table S4. Anisotropic displacement parameters (Å^2) for Li_9AlP_4 from SC-XRD measurement.

Atom	U_{11}	U_{22}	U_{33}	U_{12}	U_{13}	U_{23}
Al1	0.0081(10)	0.0081(10)	0.0081(10)	0.00000	0.00000	0.00000
Al2	0.0092(8)	0.0040(8)	0.0092(8)	0.00000	0.00000	0.00000
P1	0.0068(4)	0.0068(4)	0.0068(4)	0.0004(2)	0.0004(2)	0.0004(2)
P2	0.0081(4)	0.0068(3)	0.0074(4)	-0.0001(3)	0.0005(2)	-0.0017(3)
Li2	0.004(12)	0.021(13)	0.021(13)	0.00000	0.00000	0.00000
Li3	0.018(5)	0.018(5)	0.018(5)	0.000(3)	0.000(3)	0.000(3)
Li4	0.034(7)	0.019(6)	0.030(7)	0.00000	0.00000	0.005(6)
Li5	0.032(7)	0.037(5)	0.010(4)	0.003(4)	-0.002(3)	0.003(2)
Li6a	0.015(5)	0.015(5)	0.014(5)	0.010(4)	0.007(4)	0.003(4)
Li6b	0.017(11)	0.006(10)	0.020(12)	0.000(8)	-0.014(8)	0.003(7)
Li7	0.015(10)	0.015(10)	0.015(10)	-0.001(6)	-0.001(6)	-0.001(6)

Table S5. Selected interatomic distances in Li_9AlP_4.

atom pair			*d* / *Å*	atom pair			*d* / *Å*
Al1	P1	4x	2.424(2)	Li4	Li1	1x	2.89(1)
	Li4	6x	3.04(1)		Li7	2x	2.92(2)
Al2	P2	4x	2.4331(8)	Li5	Li7	1x	2.34(1)
	Li4	2x	2.9629(4)		Li6b	2x	2.41(2)
	Li6b	4x	2.96(2)		Li6a	1x	2.46(2)
	Li6a	4x	2.97(1)		P1	1x	2.54(1)

P1	Al1	1x	2.424(2)
	Li5	3x	2.54(1)
	Li4	3x	2.569(7)
	Li6b	3x	2.65(2)
P2	Al2	1x	2.4331(8)
	Li5	1x	2.507(9)
	Li1	1x	2.5535(7)
	Li6a	1x	2.56(1)
	Li5	1x	2.57(1)
	Li2	1x	2.5810(8)
	Li6b	1x	2.58(2)
	Li6a	1x	2.61(1)
	Li6b	1x	2.62(1)
	Li3	1x	2.63(1)
	Li4	1x	2.631(7)
	Li6a	1x	2.63(1)
Li1	Li6a	4x	2.43(1)
	P2	4x	2.639(4)
	Li4	2x	2.89(1)
	Al2	2x	2.9629(4)
	Li2	2x	2.9629(4)
Li2	P2	4x	2.5810(8)
	Li6b	4x	2.91(2)
	Li1	2x	2.9629(4)
Li3	P1	1x	2.57(3)
	P2	3x	2.63(1)(4)
	Li6a	3x	2.90(1)
	Li5	3x	2.91(3)
Li4	P1	2x	2.569(7)
	P2	2x	2.631(6)
	Li6b	2x	2.84(2)
	Li6a	2x	2.84(1)
	Li5	2x	2.85(1)
	Li6a	1x	2.56(1)
	P2	1x	2.56(1)
	P2	1x	2.734(9)
	Li4	1x	2.851(1)
	Li3	1x	2.91(3)
Li6a	Li1	1x	2.43(1)
	Li5	1x	2.46(2)
	P2	1x	2.56(1)
	Li5	1x	2.56(1)
	P2	1x	2.61(1)
	P2	1x	2.63(1)
	Li4	1x	2.84(1)
	Li6a	2x	2.90(2)
Li6b	Li5	2x	2.41(2)
	Li5	1x	2.53(2)
	P2	1x	2.58(2)
	P2	1x	2.62(2)
	P1	1x	2.65(2)
	Li4	1x	2.84(2)
	Li2	1x	2.91(2)
	Li6b	2x	2.92(4)
Li7	Li5	3x	2.34(1)
	P2	3x	2.71(1)
	Li4	3x	2.92(2)

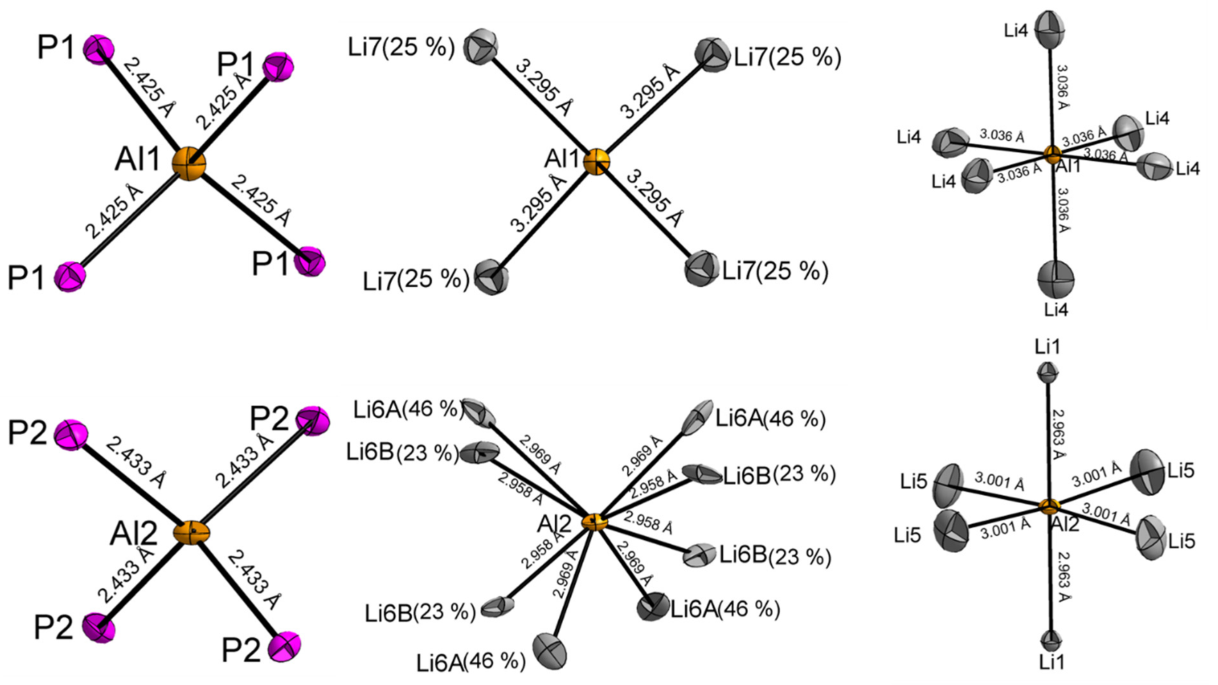

Figure S1. Coordination polyhedra of Al atoms in Li_9AlP_4. In the first coordination sphere the neighbours form slightly distorted tetrahedra and in the second coordination sphere slightly distorted octahedra.

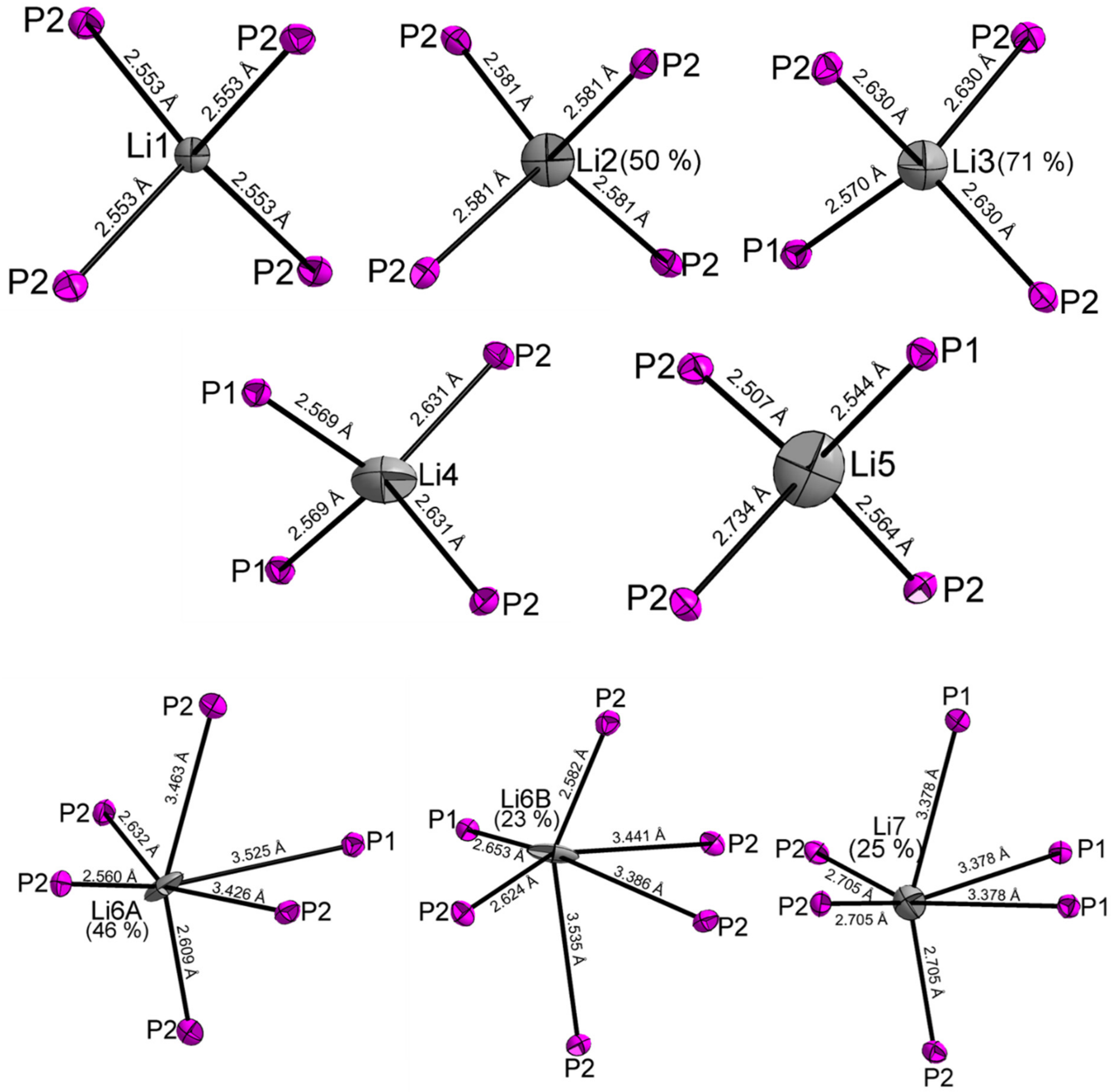

Figure S2. Coordination polyhedra of Li atoms in Li_9AlP_4. In the first coordination sphere the neighbours form slightly distorted tetrahedra and in the second coordination sphere strongly distorted octahedra.

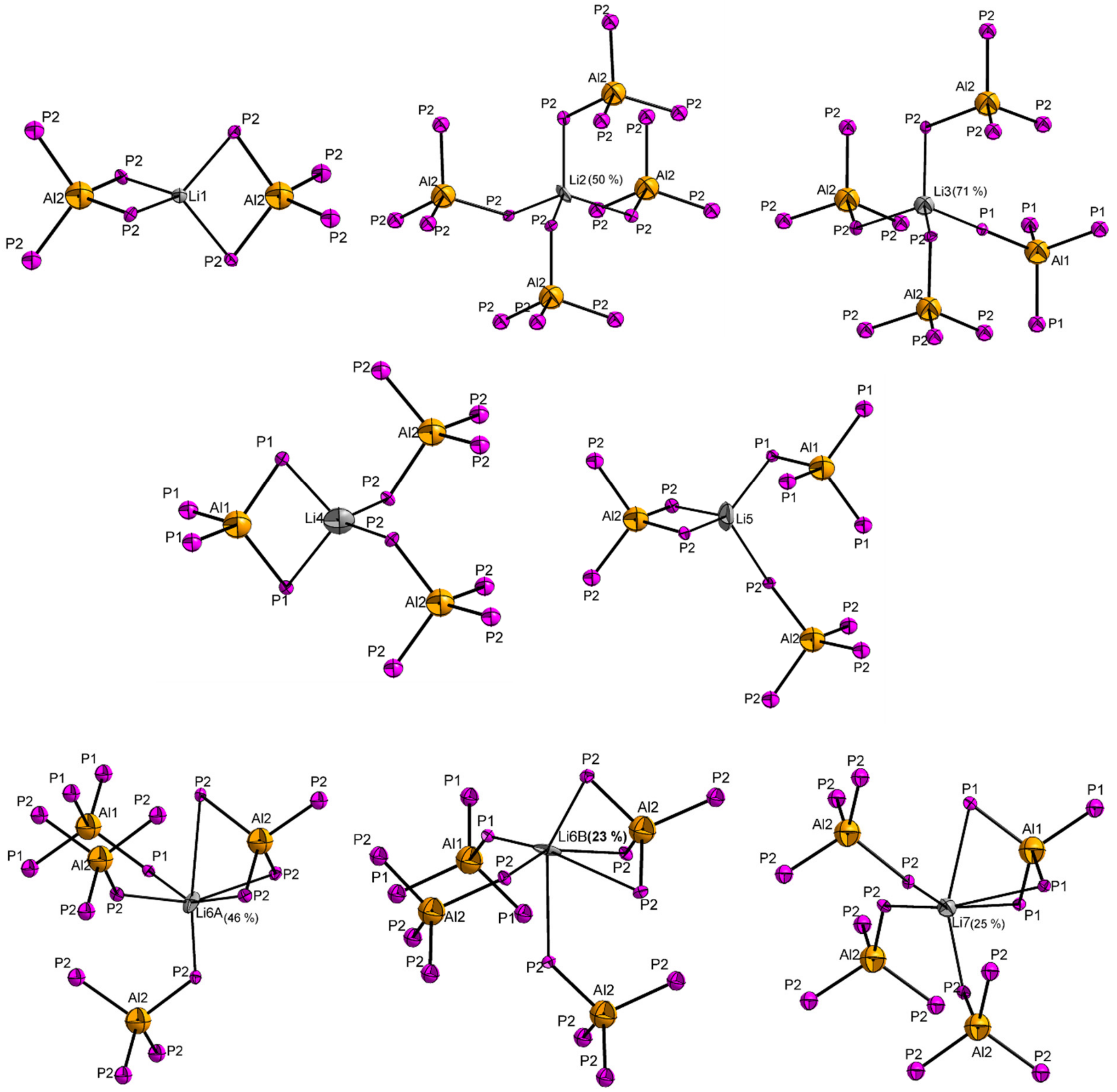

Figure S3. Li atoms with adjacent AlP_4 tetrahedra. Every tetrahedral LiP_4 tetrahedra has a different AlP_4 surroundings. Li1: two AlP_4 tetrahedra edge shared, Li2: four AlP_4 tetrahedra tetrahedral coordinated, Li3: three AlP_4 tetrahedra tetrahedral coordinated and one AlP_4 tetrahedron linear coordinated, Li4: two AlP_4 tetrahedra tetrahedral coordinated and one AlP_4 tetrahedron edge shared, Li5: one AlP_4 tetrahedra tetrahedral coordinated, one AlP_4 tetrahedron linear coordinated and one AlP_4 tetrahedron edge shared. The LiP_6 octahedra are surrounded by three AlP_4 tetrahedra tetrahedral coordinated and one AlP_4 tetrahedron face shared.

EDX Measurements

Table S6. Al/P ratio from EDX measurements for Li_9AlP_4 in comparison with the theoretical ratio from the nominal formulas. Regarding the experimental Al/P ratio from EDX the calculated formula would be $Li_{8.4}AlP_{3.8}$ ≡ $Li_{8.85}Al_{1.05}P_4$ assuming charge balance.

Atom $\%_{exp}$ Al	Atom $\%_{exp}$ P	Atom $\%_{theo}$ Al	Atom $\%_{theo}$ P
20.8(8)	79.2(8)	20	80

PXRD Measurements

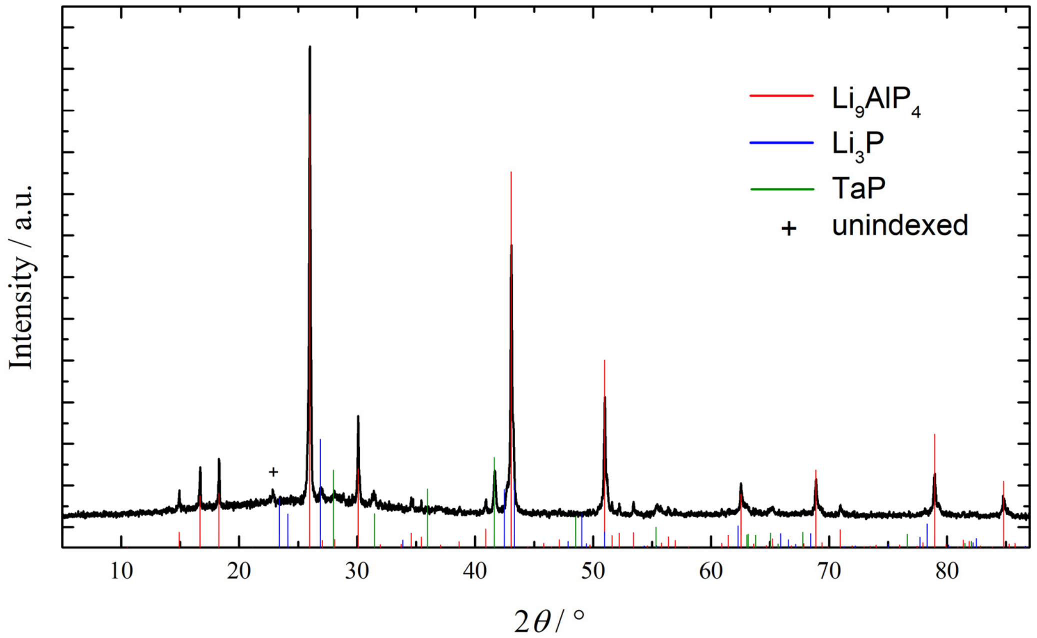

Figure S4. Experimental powder X-ray diffraction pattern of the product of the reaction 9 Li + Al + 4.2 P at 1073 K.

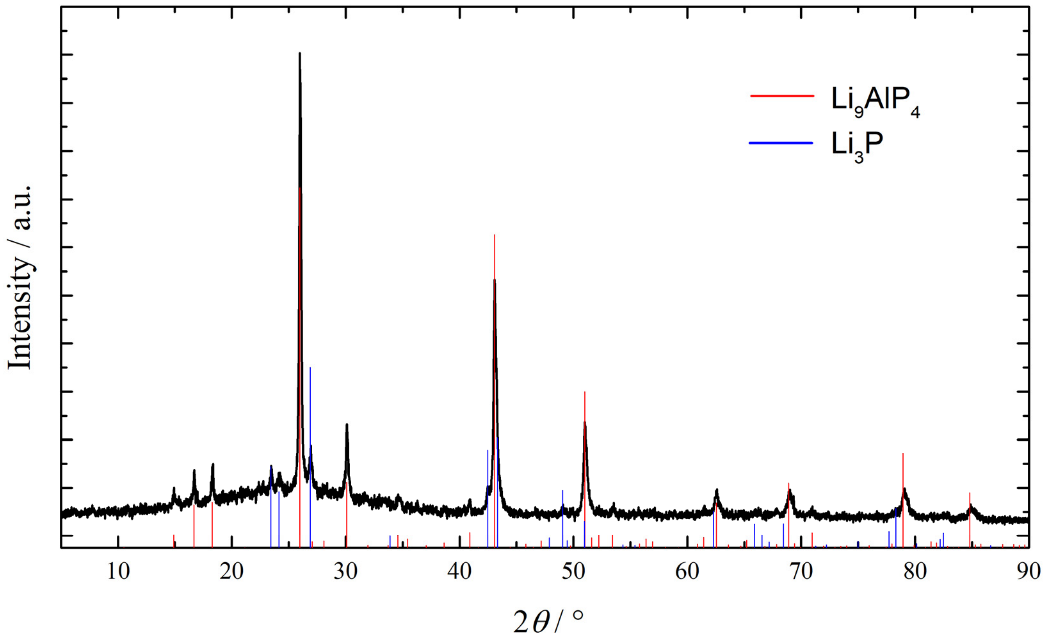

Figure S5. Experimental powder X-ray diffraction pattern of the product of the reaction 3 Li_3P + Al + P at 1073 K.

Ternary Phase System

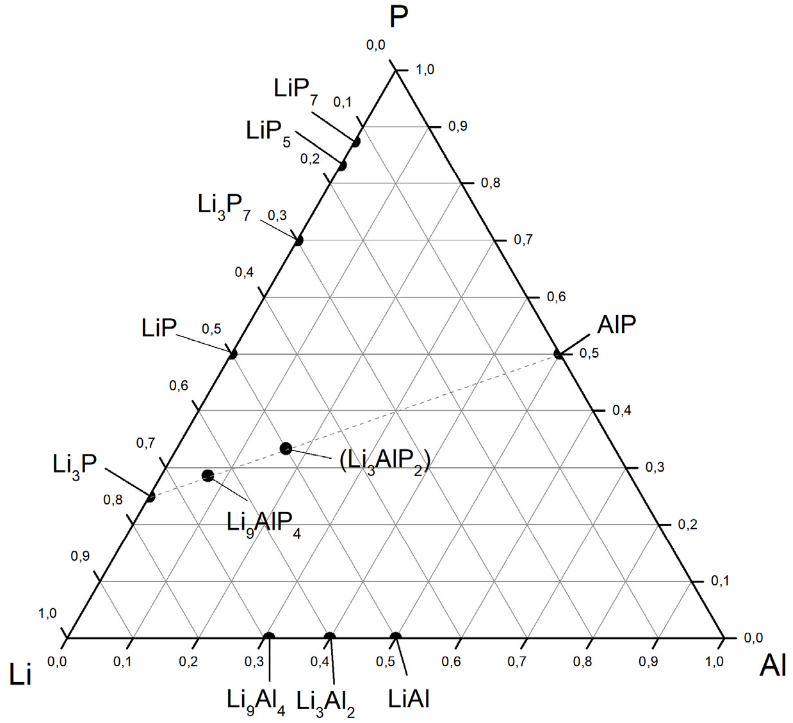

Figure S6. Ternary phase system of Li–Al–P. Li_3AlP_4 is written in brackets due to insufficient characterization by Juza.[8]

Impedance measurement procedure and examination of cell tightness

The impedance analysis approach in this work comprises two different measurement types for each cell. First, lithium ion conductivity at room temperature was measured inside an Ar filled glovebox. Secondly, the cell was taken out of the glovebox and temperature dependent measurements were performed in a climate chamber in order to determine E_A^{PEIS}. Thereby, one cycle comprises heating the cell from 298 K to 353 K and a subsequent cool down to 273 K. During a cycle, the impedance was measured two times each at 298, 313 and 333 K (once during heating and once during cooling) and one time each at 353 and 273 K, as shown in Figure S6a. One complete measurement comprises four experimental steps: cycle 0, which is the measurement at 299 – 300 K inside the glovebox, followed by cycles 1, 2 and 3, which are temperature dependent measurements outside the glovebox according to the described temperature ramp. The quality of the sealing of the cell against ambient air was evaluated by comparing ionic conductivities of cycle 0 taken inside the glovebox with the first 298 K measurement points of cycles 1–3 taken outside the glovebox, marked by the red diamonds in Figure S6a. Comparing the thus obtained conductivities, a decrease in conductivity is observed when operating the cell outside the glovebox (≈ 23% over the course of ≈ 34 h; see Figure S6b), presumably due imperfect cell sealing against ambient air which might lead to the decomposition reactions of the

solid electrolyte with ambient air and also due to a slight temperature difference from the measurement inside the glovebox (299 – 300 K) to the measurements in the climate chamber (298 K). Hence, for determining E_A^{PEIS} only cycle 1 of three independent measurements was used. In this case, the experimental error in the conductivity due to cell leakiness was estimated to be approximately 19%, compared to measuring under inert gas atmosphere.

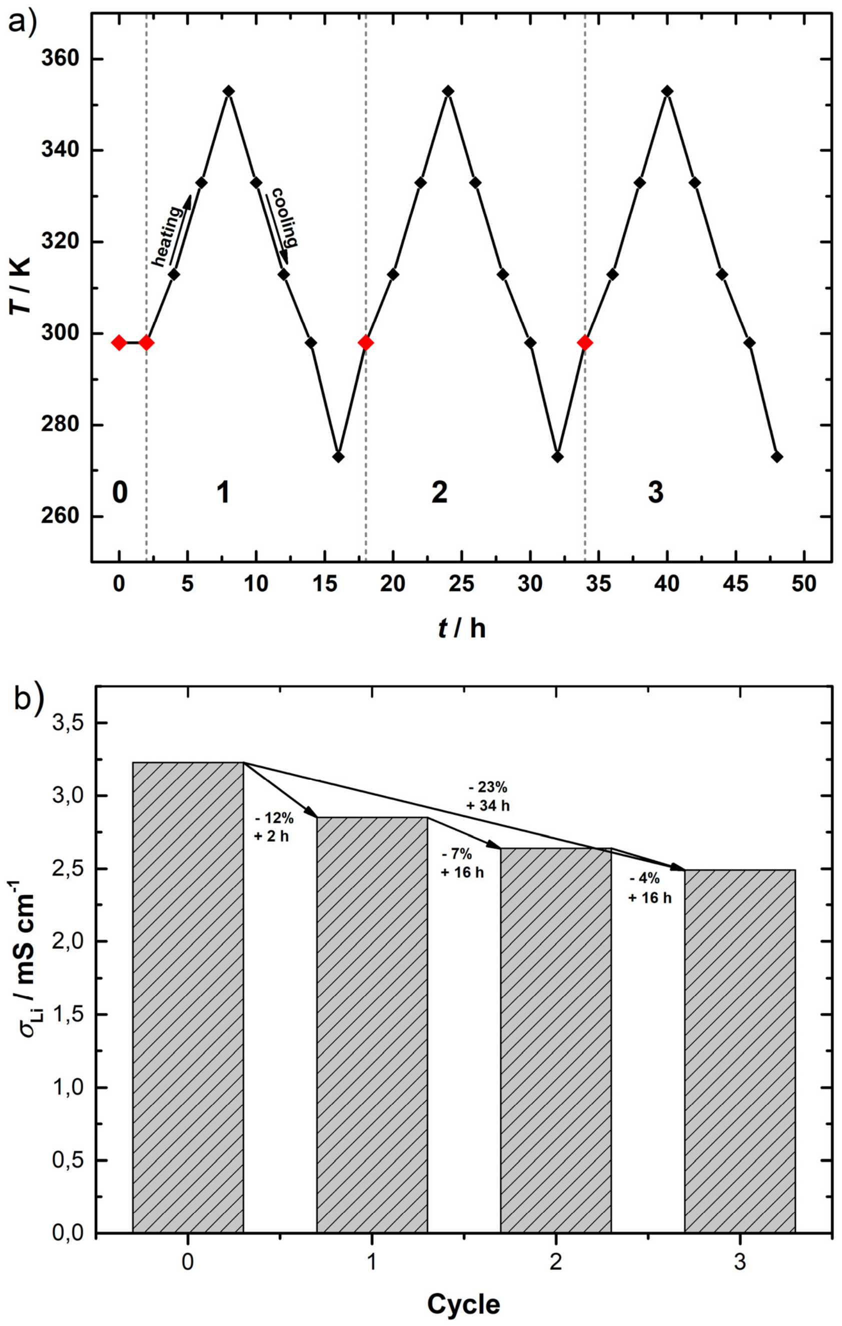

Figure S7. a) Temperature versus time profile of the lithium ion conductivity of Li_9AlP_4 in order to assess the sealing quality of the cell against ambient air. Black diamonds represent impedance measurements during heating and cooling cycles, red diamonds mark the points taken at 298 K, either still within the Ar-filled glovebox (299 – 300 K, cycle 0) or at the beginning of subsequent temperature cycles (cycles 1-3) during which the cell is exposed to ambient air. Individual cycles are indicted by numbers and separated by dashed lines. b) Lithium ion conductivity progression from cycle 0-1 (at 298 K), including the relative conductivity loss from cycle to cycle over the specified amount of time.

First-principles modelling

Computations based on density-functional theory (DFT) were carried out to supplement the experimental observations. Due to the presence of fractional site occupation factors in the refinement result (Table S3), it is required for DFT modelling to construct approximate structural models in which the (discrete) site occupations are chosen such as to mimic the experimental observations as well as possible while keeping the computational effort reasonable.

Our simulations are based on a slightly simpler structural model, derived from a refinement of single-crystal XRD data in which besides Li1, Li4, and Li5, the Li2 position had been fully occupied as well, and in which the refined composition is $Li_{9.01}AlP_4$. We proceeded as follows:

(i) Create a simulation cell using experimental lattice parameters, the fully occupied Al and P positions (**Figure S8a**), and the fully occupied lithium positions Li1, Li2, Li4, and Li5.

(ii) Randomly occupy 4 of 6 Li3 positions (≡ 67%) and 2 of 8 Li7 positions (≡ 25%) in the cell. This distribution provides a reasonable approximation to the experimental s.o.f. values underlying our model, which are 0.60(5) and 0.30(4), respectively; it comes even closer to the final refinement result of 0.71(4) and 0.25(4) (Table S3). This specific set of initial positions is the same in all models in the following. The result is shown in **Figure S8b**, which also includes the above-mentioned fully occupied Li positions.

(iii) Occupy the remaining lithium positions, Li6a and Li6b—which are of largest interest here because they both are found in the same octahedron, each with fractional occupations (Figure 2c of the main text). 16 atoms were distributed in this way, in good approximation of the combined experimental occupancy on these sites (Table S3). The refinement result indicates a preference for Li6a (Table S3), and **Figure S8c** shows a randomly occupied model that approximates this distribution. We ultimately decided to create randomized structural models in which *only* Li6a or *only* Li6b positions are occupied; static computations (without relaxation) for these qualitatively corroborate the preference for Li6a (Figure 3a of the main text).

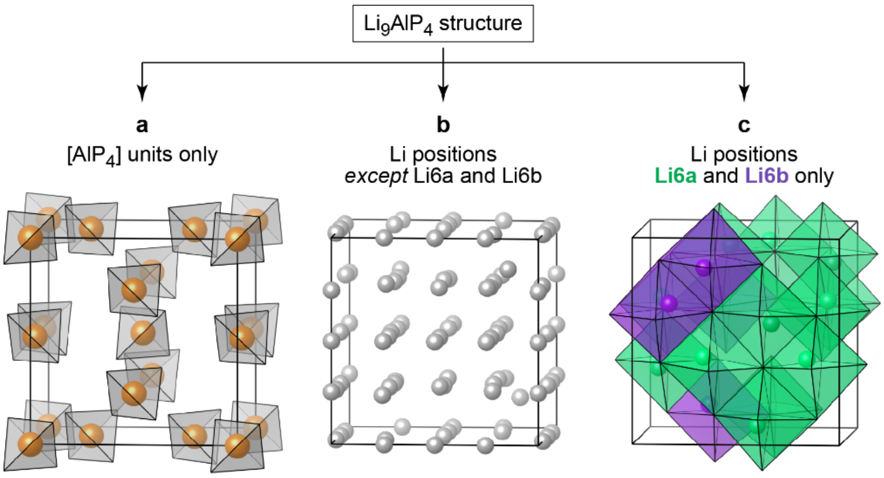

Figure S8. The conceptual approach for modelling the Li_9AlP_4 structure in DFT computations, which requires a discrete occupation model for the disordered sites. Three structural drawings are presented, each showing parts of the atoms in the same structure, prior to DFT relaxation. (a) Al and P positions only. These are all fully occupied and lead to a Cr_3Si-like network of tetrahedra, between which Li atoms are inserted in the next steps. (b) The fully occupied Li positions as well as Li3 and Li7. For the latter, we take some of the sites to be occupied (assigned randomly), roughly in line with the experimental sof (Table

S3). The occupation of these sites is kept fixed in all subsequent computations. (c) The Li6a and Li6b positions, residing within octahedral voids of the ccp arrangement of P atoms.

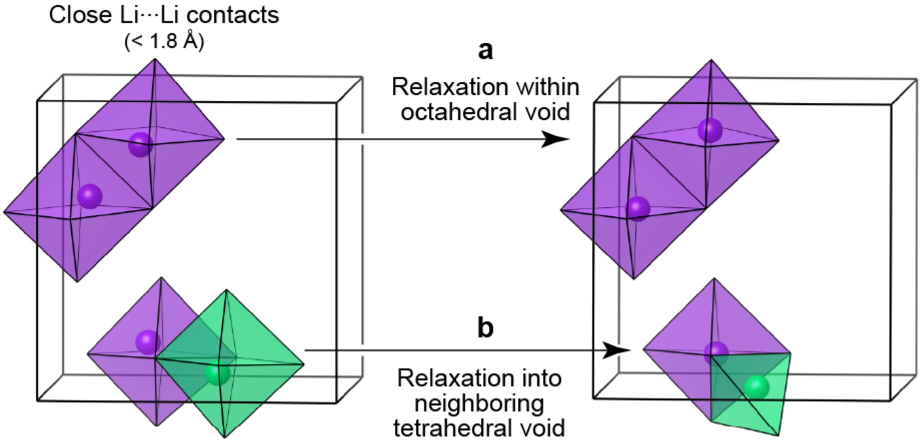

Figure S9. Qualitative relaxation mechanisms, showing examples for the DFT relaxation of the mixed structural model shown in Figure S8c (the left-hand side structure uses experimentally derived atomic coordinates; the right-hand side one has been relaxed with DFT). (a) Close Li-Li contacts resulting from two neighbouring Li6b sites (purple) being occupied. Upon relaxation, the atoms are pushing each other apart, thus leaving the Li6b position but remaining within the respective octahedral void. (b) An atom (initially assigned to a Li6a position; green) moving into a neighbouring tetrahedral void.

DFT-based structural relaxations were performed for these models; the resulting energies for the above-mentioned ten structural models have been visualized in Figure 3a of the main text, and the corresponding numerical values are given in **Table S7**. Upon relaxation, some Li atoms change their position (**Figure S9**) whereas the "framework" of [AlP_4] units remains intact. This is consistent with the notion of highly mobile Li ions in the structure, also resembling what we observed in our recent report on $Li_{14}SiP_6$ (see main text). Due to the complex structural rearrangements and the occasional relaxation of an Li atom into a neighbouring position, the initial assignment only to Li6a or only to Li6b positions is no longer valid in the relaxed structural models. Concomitantly, the ten optimized systems are practically indistinguishable in energy (see Figure 3a of the main text).

Table S7. Energetics, as obtained from DFT, of ten structural models (see Figure S8), in which either the Li6a position (index 1–5) or the Li6b position (6–10) is initially occupied. "LAP" indicates the ternary compound; energies of binary phases are obtained for fully DFT-optimized structures (a = 5.468 Å for AlP; data for Li_3P in Table S8); see text.

		E(LAP; unrelaxed)	*E*(LAP; relaxed)	*E*(LAP; relaxed) − [3 *E*(Li_3P) + *E*(AlP)]	
	Index	(eV / f.u.)	(eV / f.u.)	(eV / f.u.)	(kJ / mol)
Li6a occupied	1	−2801.813638	−2803.318525	−0.38	−36.2
	2	−2801.774613	−2803.285850	−0.34	−33.1
	3	−2801.654988	−2803.238500	−0.30	−28.5
	4	−2801.566813	−2803.232663	−0.29	−28.0
	5	−2801.405700	−2803.294313	−0.35	−33.9
Li6b occupied	6	−2800.659550	−2803.294850	−0.35	−34.0
	7	−2800.589725	−2803.245300	−0.30	−29.2
	8	−2800.581000	−2803.217175	−0.27	−26.5
	9	−2800.552150	−2803.182250	−0.24	−23.1
	10	−2800.522363	−2803.271725	−0.33	−31.7

We also optimized the structures of the elemental and binary phases. The accuracy of the specific DFT methodology we use (see below) can be assessed by comparing the computed structural parameters for the compositionally closely related Li_3P with an accurate experimental reference (Table S8). We use the binaries as a reference, as per the formal reaction

$$3\ Li_3P + AlP \rightarrow Li_9AlP_4\ ;$$

and the energy difference associated with this reaction indicates stability of the ternary compounds (right-hand side of Table S7). We note the following limitations to our stability analysis: (i) we do not take configurational enthalpy into account, which will stabilize the ternary but not the binary phases at finite temperature; (ii) we keep the lattice parameters of the ternary model systems fixed to the initial settings during the optimization, whereas the structures of the binaries are fully optimized. If those limitations were relaxed, the ternary phase might become even more favourable with respect to the binaries.

Table S8. Structural parameters of Li_3P. Experimental data from single-crystal X-ray diffraction at 167 K Space group $P6_3/mmc$; Li1 and P on 2*b* and 2*c*, respectively, without free parameters.

	Expt. (Dong & DiSalvo)	DFT (this work)
a (Å)	4.2286(3)	4.1986
c (Å)	7.5566(13)	7.5034
V (Å^3)	117.0	114.6
Li2 on 4*f* (1/3, 2/3, *z*)	*z* = 0.5839(4)	*z* = 0.5841

We also estimate the energies of formation by referencing the computed energies for the binary and ternary phases to DFT-optimized elemental structures, *viz.* bcc Li, ccp Al, and black P (**Table S9**). The average values and standard deviations given for the ternary models correspond to the ten optimized structures listed in Table S6.

Table S9. Computed energies of formation (with respect to the elements). The ternary compound is more stable than the competing binaries by ≈ 30 kJ / mol.

	ΔE_f (kJ / mol)
Li_3P	–272
AlP	–111
3 Li_3P + AlP	–928
Li_9AlP_4 (randomized)	–958(4)

Computational details

DFT computations were carried out with CASTEP 8.0[6] and on-the-fly pseudopotentials, using a two-point steepest-descent optimizer[9] for structural optimization. Reciprocal space was sampled on grids of 2×2×2 points. The cut-off energy for electronic energies was 1000 eV; the electronic convergence criterion was $\Delta E < 10^{-7}$ eV. Exchange and correlation were treated using the PBEsol functional.[7]

References

[1] H. Eickhoff, L. Toffoletti, W. Klein, G. Raudaschl-Sieber, T. F. Fässler, *Inorg. Chem.* **2017**, *56*, 6688-6694.

[2] *WinXPOW,* 3.0.2.1, STOE & Cie GmbH, Darmstadt, Germany, **2011**.

[3] *TOPAS,* 6, Bruker AXS, **2016**.

[4] G. Sheldrick, *Acta Crystallogr., Sect. C: Struct. Chem.* **2015**, *71*, 3-8.

[5] S. Strangmüller, H. Eickhoff, D. Müller, W. Klein, G. Raudaschl-Sieber, H. Kirchhain, C. Sedlmeier, V. Baran, A. Senyshyn, V. L. Deringer, L. van Wüllen, H. A. Gasteiger, T. F. Fässler, *J. Am. Chem. Soc.* **2019**.

[6] S. J. Clark, M. D. Segall, C. J. Pickard, P. J. Hasnip, M. J. Probert, K. Refson, M. C. Payne, *Z. Krist.* **2005**, *220*, 567-570.

[7] J. P. Perdew, A. Ruzsinszky, G. I. Csonka, O. A. Vydrov, G. E. Scuseria, L. A. Constantin, X. Zhou, K. Burke, *Phys. Rev. Lett.* **2008**, *100*, 136406.

[8] R. Juza, W. Schulz, *Z. Anorg. Allg. Chem.* **1952**, *269*, 1-12.

[9] J. Barzilai, J. M. Borwein, *IMA J. Numer. Anal.* **1988**, *8*, 141; CASTEP keyword "geom_method: tpsd".
